# Combined Aberrant Expression of NDRG2 and LDHA Predicts Hepatocellular Carcinoma Prognosis and Mediates the Anti-tumor Effect of Gemcitabine

**DOI:** 10.7150/ijbs.35094

**Published:** 2019-07-03

**Authors:** Yan Guo, Xi'an Li, Xiang Sun, Jiancai Wang, Xu Yang, Xin Zhou, Xinping Liu, Wenchao Liu, Jianlin Yuan, Libo Yao, Xia Li, Lan Shen

**Affiliations:** 1 The State Key Laboratory of Cancer Biology, Department of Biochemistry and Molecular Biology, The Fourth Military Medical University, Xi'an, Shaanxi, 710032, China;; 2 Department of Prosthodontics, School of Stomatology, The Fourth Military Medical University, Xi'an, Shaanxi, 710032, China; 3 Department of Oncology, State Key Discipline of Cell Biology, Xijing Hospital, The Fourth Military Medical University, Xi'an, Shaanxi, 710032, China;; 4 Department of Urology, Xijing Hospital, The Fourth Military Medical University, Xi'an, Shaanxi, 710032, China;

**Keywords:** Warburg effect, NDRG2, LDHA, gemcitabine, hepatocellular carcinoma

## Abstract

The Warburg effect is one of the important hallmarks of cancer. The activation of oncogene and inactivation of tumor suppressor gene contribute to the enhancement of glycolytic enzymes and the Warburg effect. The N-myc downstream regulated gene 2 (NDRG2) is a tumor suppressor gene and is frequently lost in various types of cancer. However, little is known about glycolytic function and therapeutic value of NDRG2 in hepatocellular carcinoma (HCC). In this study, we found that NDRG2 and lactate dehydrogenase A (LDHA) were aberrantly expressed in HCC and were closely related to the Warburg effect. The correlation between NDRG2 and LDHA expression predicted HCC prognosis and the clinical response to chemotherapy. NDRG2 expression was significantly decreased while LDHA expression was increased in HCC specimens. NDRG2 and LDHA expression was significantly correlated with differentiation status, vascular invasion, and TNM stage of HCC. NDRG2 inhibited LDHA expression, the Warburg effect and the growth of HCC cells. Furthermore, NDRG2 mediated gemcitabine-induced inhibition of LDHA expression and the Warburg effect in HCC cells. Taken together, our data suggest that NDRG2 plays an important role in inhibiting the Warburg effect and the malignant growth of HCC via LDHA. NDRG2 combined with LDHA might be powerful prognostic biomarkers and targets for chemotherapy treatment of HCC.

## Introduction

Hepatocellular carcinoma (HCC) is one of the most common malignancies worldwide, accounting for 85% to 90% of primary liver cancers 1, 2. The standard HCC treatments include surgical resection, liver transplantation, immunotherapy, radiotherapy and chemotherapy 3, 4. However, none of these treatments is very effective, and a large proportion of HCC patients have a poor prognosis because of rapid metastasis 4, 5. Therefore, there is an urgent need for other treatment modalities, such as molecular targeted therapy.

N-Myc downstream-regulated gene 2 (NDRG2), a member of the NDRG family, was firstly discovered by our laboratory using subtractive hybridization 6. Our previous research revealed that DNA damage, hypoxia, and glucocorticoids promoted NDRG2 expression and NDRG2 can be transcriptionally activated by p53 and HIF1α 7-9. More importantly, NDRG2 is closely related to the occurrence and development of hepatocellular carcinoma. NDRG2 expression was negatively correlated with malignancy in HCC and inhibited the proliferation and invasion of HCC 10, 11. Furthermore, NDRG2 inhibits HCC invasion and migration as well as opposes HCC progression 12. As a tumor suppressor, NDRG2 inhibited glycolysis and glutaminolysis in colorectal cancer cells 13. Moreover, NDRG2 also inhibited the activation of fatty acid oxidation under glucose limitation in hepatoma cells 11. Therefore, NDRG2 is an important suppressor in tumor metabolic reprogramming.

Lactate dehydrogenase A (LDHA), which can catalyze pyruvate into lactate, plays many roles in non-neoplastic and neoplastic cell. LDHA is involved in glycolysis, gene transcription, cell cycle regulation, and brain development 14. LDHA is aberrantly elevated in many types of cancers and acts as a key checkpoint of aerobic glycolysis in cancer cells. LDHA is upregulated in multiple types of cancers and promotes metabolic reprogramming and malignant proliferation of cancer cells 15. The expression of LDHA can be regulated by many factors, such as transcription factors, transcriptional coactivators, and protein kinases 16. In addition, LDHA is an important glycolytic enzyme that promotes the growth and metastasis of hepatocellular carcinoma 17. Recently, several patents on LDHA small-molecule inhibitors were reported 18. For example, LDHA inhibitor quinolone 3-sulfonamides can reverse aerobic glycolysis in cancer cells 19. Therefore, LDHA is a potential therapeutic target for hepatocellular carcinoma. The diagnostic values and regulatory mechanisms of LDHA in HCC need to be further investigated.

Gemcitabine is commonly used for solid tumor chemotherapy. Gemcitabine plus oxaliplatin is effective and safe in patients with advanced HCC 20. The molecular mechanism of cell proliferation inhibition induced by gemcitabine is complex. A previous study has shown that novel LDHA inhibitors have synergistic cytotoxic activity with gemcitabine against pancreatic cancer cells, maybe there is some correlation between Gemcitabine and LDHA 21. In addition to inhibition of the synthesis of nucleotides, the effect and mechanism of gemcitabine on nutrition and energy metabolism need to be further explored.

Herein, we provide evidence that NDRG2 is significantly decreased while LDHA is increased in HCC. The expression levels of NDRG2 and LDHA were closely correlated with outcome of HCC patients. Moreover, NDRG2 inhibited LDHA expression, aerobic glycolysis, and cell proliferation and mediated the effect of gemcitabine chemotherapy.

## Materials and Methods

### Study cohort and tissue samples

This study protocol was performed in accordance with the guidelines outlined in the Declaration of Helsinki and was approved by the Ethics Committee of the Fourth Military Medical University. Written informed consent was obtained from all participants. The hospital-based study cohort included 140 patients that were randomly selected from patients diagnosed with HCC in Xijing Hospital, Fourth Military Medical University. The clinicopathological information and follow-up data of the patients were recorded into a database that was under a close follow-up scheme and updated with respect to survival status by telephone visit and questionnaire letters. Normal liver tissues that were obtained from patients who underwent surgery without malignancy served as controls. All the fresh tissues were obtained within 10 minutes and then placed into liquid nitrogen for mRNA and protein preparation. All the specimens were histologically diagnosed by the Department of Pathology, Xijing Hospital, Fourth Military Medical University. Study physicians who reviewed and recorded all the medical records of HCC patients were completely blind to the exposure data. Progression-free survival was measured from the date of treatment to the time of disease progression. Overall survival was measured from the date of treatment to the time of death or censoring.

### Cell culture and materials

The human liver cancer cell lines HuH-7, HepG2, HHCC, MHCC97L and MHCC97H as well as the human normal liver cell line QSG-7701 were used in the present study. The HepG2 cells were from ATCC; MHCC97L cells were from Fudan University; QSG-7701, HuH-7 and MHCC97H cells were from the cell bank of the Chinese Academy of Sciences. HHCC cells was firstly purchased from Shanghai Institute of Cell Biology, Chinese Academy of Sciences (Shanghai, China) and stocked in our lab. HHCC cells were widely used as hepatocellular carcinoma cells in many research reports 22-24. Cells were maintained in the medium recommended and supplemented with 10% FBS in a 37°C and 5% CO_2_ incubator. Recombinant lentiviral vectors were constructed with an Invitrogen ViraPower^TM^ Lentiviral System (Carlsbad, CA, USA) in our laboratory 13. The lentiviral vectors pLenti6-mCherry/NDRG2, pLKO-Scramble/NDRG2-shRNA, PAX2 and PMD2G were transfected into HEK-293T cells using Lipofectamine 2000 (Invitrogen) according to the manufacturer's instructions. HCC cells were infected with the viral medium from HEK-293T cells 48 h after transfection.

### Immunohistochemistry

The tissue microarray (TMA) staining was performed using standard immunohistochemistry procedures. The slides were incubated overnight with primary antibodies against NDRG2 (Abnova, Taipei, Taiwan) or LDHA (Cell Signaling, Danvers, MA, USA). Mayer's hematoxylin was used for the purpose of nuclear counter-staining. In this study, the number of positively stained cells and the intensity of positive staining on liver cells were independently scored by two pathologists in a blinded manner. The extensional immunoreactivity score standard incorporated (1) the number of cells with positive staining (≤ 5%: 0; 6-25%: 1; 26-50%: 2; 51-75%: 3; and >75%: 4) and (2) the staining intensity (colorless: 0; pale yellow: 1; yellow: 2; brown: 3). The staining grade was stratified as absent (0 score), weak (1-4 score), moderate (5-8 score) or strong (9-12 score).

Tumor tissues from nude mice were collected on day 28, excised and fixed with 4% formalin, and embedded in paraffin. For immunohistochemistry, 5 µm-thick tissue sections were cut, dewaxed in xylene, and rehydrated. To perform Ki67 staining, the slides were incubated with 1% bovine serum albumin in PBS at room temperature for 1 h for blocking and then stained with primary antibodies against NDRG2 (Abnova, Taipei, Taiwan), LDHA (Cell signaling, Danvers, MA, USA) or Ki-67 (Neomarkers, Fremont, CA, USA) at room temperature for 4 h. They were subsequently washed three times with PBS to remove excess primary antibody and then incubated with anti-mouse HRP-conjugated IgG (1:500 dilution) for 1 h at room temperature. Finally, the slides were washed three times, incubated with DAB peroxidase substrate (Sigma, St Louis, MO, USA) and covered with glass cover slips. The staining results were observed with a bright field microscope.

### Quantitative real-time PCR and Western blotting analysis

Total RNA was isolated from cells using TRIzol Reagent (Invitrogen), and then complementary DNA (cDNA) was synthesized using AMV reverse transcriptase (Promega, Madison, WI, USA) according to the manufacturer's instructions. The cDNA was used as a template for quantitative real-time PCR using an ABI Prism 7500 real-time PCR instrument (Applied Biosystems, Carlsbad, CA, USA). The primers used for real-time quantitative PCR are listed in Supplementary Table 1.

For western blotting analysis, total protein was prepared from human liver cell lines and clinical hepatocellular carcinoma tissue samples. Immunoblotting was performed according to standard procedures with polyclonal rabbit anti-human LDHA, anti-human c-Myc, anti-α-Tubulin (Cell Signaling, Bedford, MA, USA), monoclonal mouse anti-human NDRG2 (Abnova, Taipei, Taiwan), and polyclonal rabbit anti-human β-actin (Biosynthesis Biotechnology, Beijing, China) antibodies.

### Glucose consumption and Lactate production

Treated cells were seeded on 6-well plates at a density of 1×10^6^ cells per well and the culture medium was changed to low glucose DMEM without phenol red (Thermo Fisher Scientific) with or without drugs. Gemcitabine and c-Myc inhibitor 10058-F4 was purchased from Selleck Chemicals (Houston, TX, USA) and dissolved in DMSO. Sodium oxamate was purchased from Sigma-Aldrich (St Louis, MO, USA) and dissolved in water. The concentrations of glucose and lactate in the culture medium were measured after incubation of cells for 24 h with Glucose Assay Kit (Sigma-Aldrich) and Lactate Assay Kit (Jiancheng Bioengineering, Nanjing, China) individually. The glucose consumption and lactate production were normalized to cell numbers. The cell numbers were calculated and analyzed using the cellometer mini bright field automated cell counter (Nexcelom Bioscience, Lawrence, MA, USA).

### Colony formation assay

Cells were seeded into 60-mm dishes at a density of 400 cells per dish. The cells were grown for 2 weeks in culture medium. Then, the colonies were fixed and stained with crystal violet.

### MTT assay

Cells were seeded into 96-well plates in triplicate at a starting density of 1×10^4^ cells/well and then treated with gemcitabine. Treated cells were washed and incubated with tetrazolium salt (MTT, 100 µg/ml; Sigma) at 37°C for 4 h. The supernatant was removed, and 150 µl of dimethyl sulfoxide (DMSO) was added to each well. The absorbance (OD) of the reaction solution at 490 nm was recorded.

### In vivo tumorigenicity assay

The animal study and experiment protocols were approved by the Institutional Laboratory Animal Center at the Fourth Military Medical University. The animals were maintained and handled in accordance with the Guidelines for Accommodation and Care of Animals. Yan Guo has the license for animal experiments. All mice were housed in standard conditions of 12-hour light/dark cycle and access to food and water ad libitum. Four-week-old athymic mice were injected subcutaneously in the left limb with 1×10^7^ cells in Hank's balanced salt solution and Matrigel (Invitrogen) mixed 1:1. Tumor growth was monitored by measuring tumor size using Vernier calipers every 3 days for a 4-week period. Tumor-bearing mice were treated intraperitoneally with 80 mg/kg gemcitabine in 200 μL normal salt solution on 8, 12 and 16 days post tumor inoculation (d.p.i.). At the end of the experiment, tumor weight was assessed by sacrificing the mice, removing and weighting the tumor.

### Statistical analysis

Statistical analysis was performed with SPSS software (version 17.0; SPSS, Chicago, IL, USA). Results are presented as mean ± standard deviation (SD) from at least three individual experiments for each group. Student's t-test was used to compare the difference of two groups. Associations between NDRG2 and LDHA IHC staining and clinicopathological variables were analyzed using Pearson's chi-square test. Spearman's rank correlation coefficient analysis was performed to assess the correlation between the expression of NDRG2 and LDHA in HCC tissues. The patient's survival analysis was evaluated using the Kaplan-Meier method, and the difference between survival curves of patients and NDRG2 or LDHA expression level was analyzed using the log-rank test. Cox proportional-hazard analysis was used for performing univariate and multivariate analysis: these kinds of analysis were used to explore the effect of variables on survivals. Statistical significance was defined as* P*<0.05, and histograms were prepared with Origin 6.0 (Microcal Software, Inc. Northampton, MA, USA).

## Results

### NDRG2 and LDHA expression correlates with clinicopathological characteristics of HCC patients

**Figure 1:**
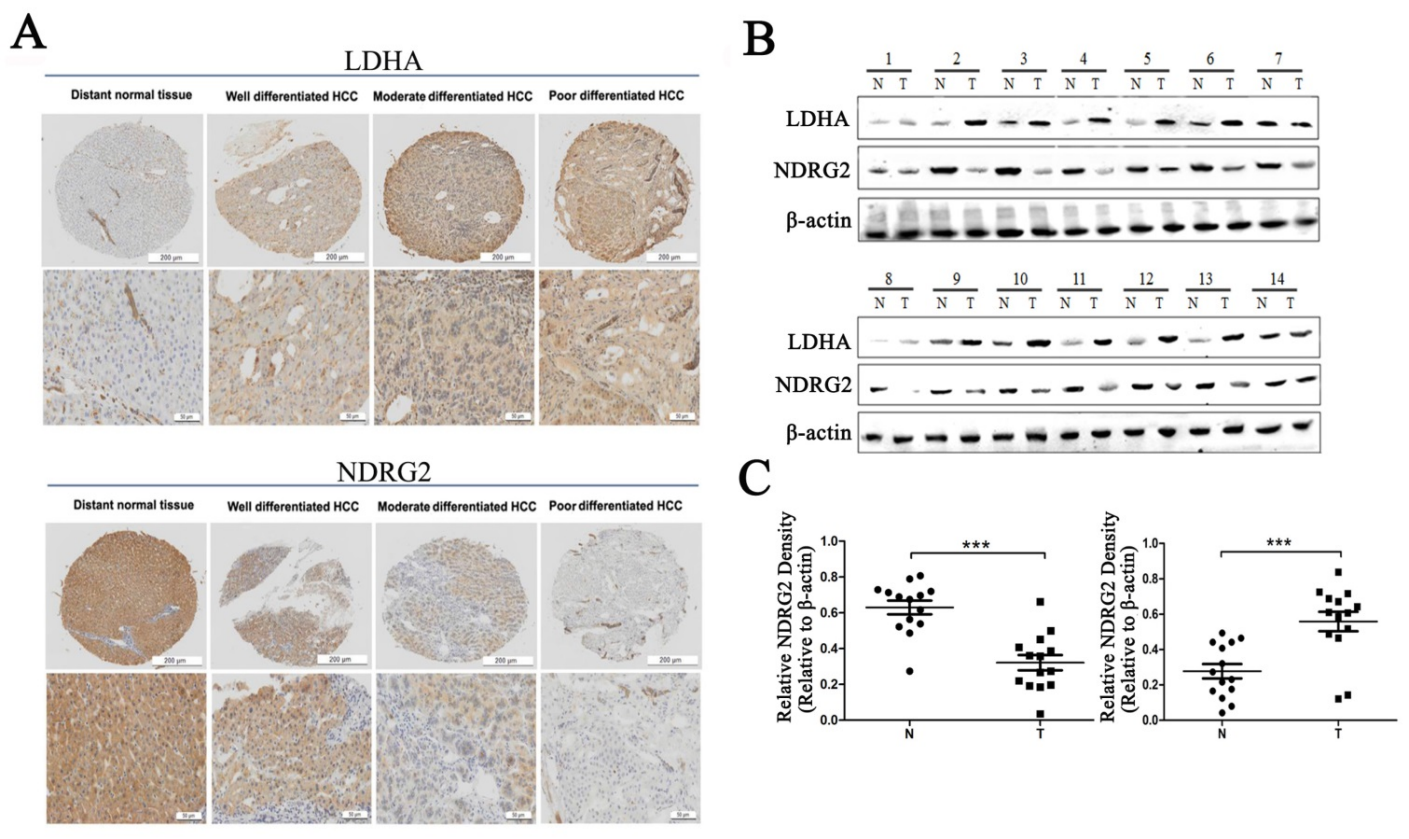
** NDRG2 and LDHA expression in liver tissues of patients with HCC. (A)** NDRG2 and LDHA immunostaining of tissue microarrays comprising 20 distant normal tissues, 27 well differentiated HCC tissues, 74 moderately differentiated HCC tissues and 39 poorly differentiated HCC tissues. **(B)** NDRG2 and LDHA protein levels in fresh HCC tissues (T) and adjacent normal tissues (N) were detected by Western blotting. **(C)** Relative quantification data of NDRG2 and LDHA protein levels in fresh HCC tissues (T) and adjacent normal tissues (N). Scale bars, 200 μm or 50 μm. ***p<0.001.

To verify the relationship between NDRG2 and LDHA expression and the clinicopathological characteristics of HCC patients, we investigated the expression of NDRG2 and LDHA in tissues from clinical HCC patients who has been histologically diagnosed between November 2010 and November 2012 by Department of Pathology, Xijing Hospital, Fourth Military Medical University. First, we analyzed the expression of NDRG2 and LDHA in tissue microarrays containing 27 well differentiated HCCs, 74 moderately differentiated HCCs, and 39 poorly differentiated HCCs using immunohistochemistry. Consistent with our previous study, NDRG2 is primarily expressed in hepatocytes 25, 26. NDRG2 protein level in poorly differentiated HCC tissues (*n*=39) was lower than that in well differentiated HCC tissues (*n*=27) and moderately differentiated HCC tissues (*n*=74). NDRG2 expression enhanced with the increase of HCC differentiation degree. In contrast, LDHA protein level in poorly differentiated HCC tissues was higher than that in well differentiated HCC tissues and moderately differentiated HCC tissues. LDHA expression decreased with the differentiation of HCC tissues (**Figure 1A**). Western blotting analysis of fresh HCC and adjacent non-carcinoma tissues from 14 patients with HCC revealed that NDRG2 decreased while LDHA increased significantly in HCC tissues compared with adjacent non-carcinoma liver tissues (**Figure 1B**), and thus, there was a negative correlation between the expression of NDRG2 and LDHA in HCC tissues (**Figure 1C**).

The correlation between NDRG2 and LDHA expression levels and different clinicopathological factors was analyzed in the above clinical HCC specimens (**Table 1**). No statistically significant correlations were observed between NDRG2 and LDHA expression and age, gender, AFP, tumor size, or HBV infection. Statistically significant correlations were found between a low level of NDRG2 expression and a poor degree of histological differentiation (*P*<0.001), the presence of vascular invasion (*P*<0.001), and advanced TNM stage (*P*<0.001). Meanwhile, statistically significant correlations were found between the high level of LDHA expression and the poor degree of histological differentiation (*P*<0.001), the presence of vascular invasion (*P*=0.024), and advanced TNM stage (*P*=0.027).

### Decreased NDRG2 and increased LDHA expression correlates with poor survival of HCC patients

**Figure 2:**
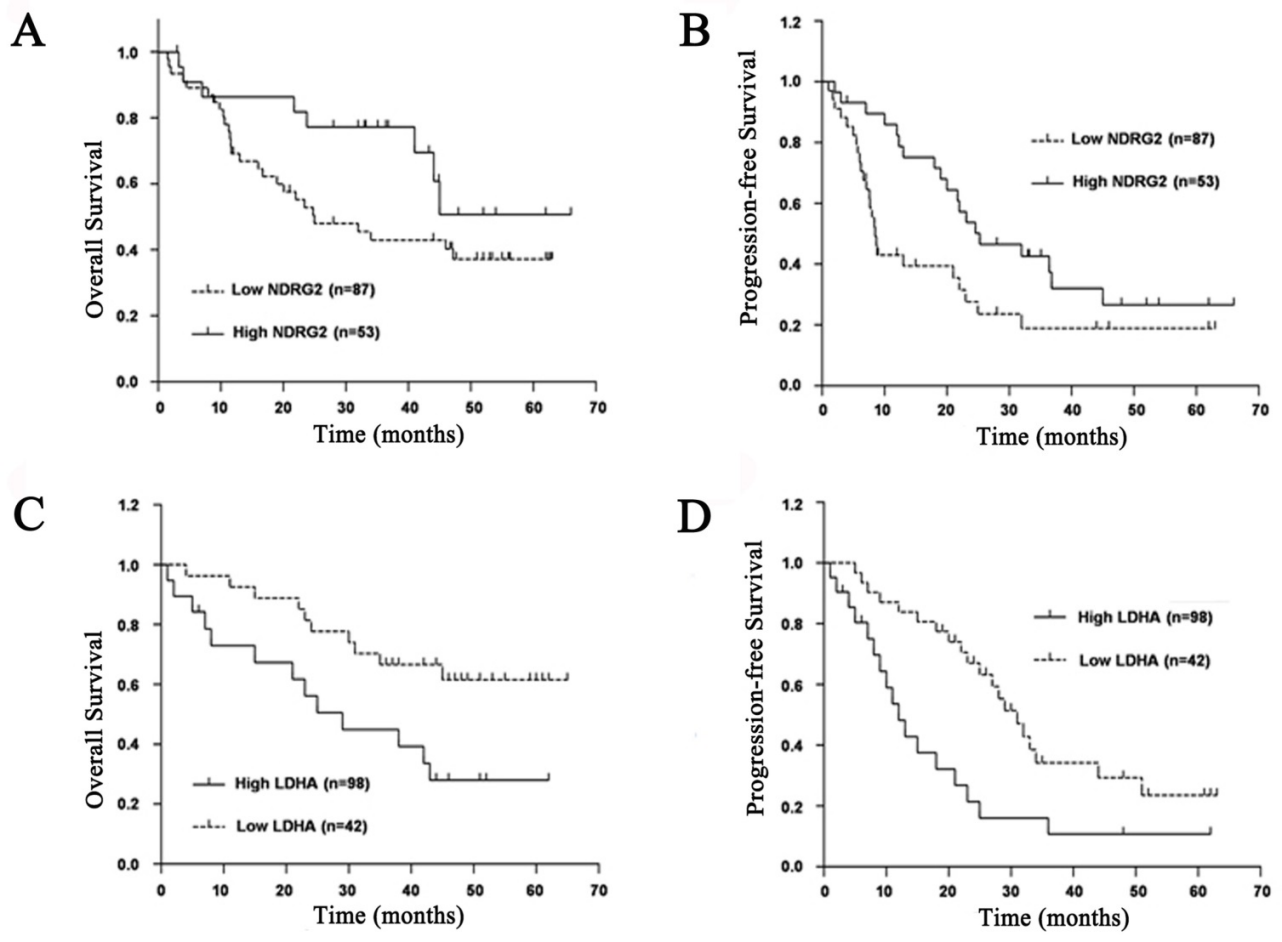
** Prognostic significance of NDRG2 and LDHA expression in patients with HCC. (A)** NDRG2 expression for overall survival. **(B)** NDRG2 expression for disease-free survival. **(C)** LDHA expression for overall survival.** (D)** LDHA expression for disease-free survival.

To evaluate prognostic factors for survival rate after comprehensive treatment of HCC patients, univariate and multivariate analyses were performed in 140 HCC patients. A statistically significant association between poor overall survival and progression-free survival and a low NDRG2 and a high LDHA expression level was found in HCC patients (**Figure 2**). The Kaplan-Meier analysis for postoperative overall survival and progression-free survival showed that HCC patients with preserved NDRG2 and reduced LDHA expression had longer overall survival and progression-free survival than patients with reduced NDRG2 and preserved LDHA expression (**Figure 2**).

The univariate analysis results showed that NDRG2 and LDHA protein levels are closely related to overall survival of HCC patients independent of age, gender, AFP, tumor size and HBsAg. Histological differentiation, vascular invasion, and TNM stage were associated with overall survival of HCC patients (**Table 2**). The multivariate analysis results showed that HCC patients with reduced NDRG2 expression had worse overall survival and higher risk of death and recrudescence than those with preserved NDRG2 expression. In contrast, HCC patients with reduced LDHA expression had better overall survival and lower risk of death and recrudescence than those with preserved LDHA expression. Therefore, NDRG2 and LDHA protein levels may be two prognostic factors for overall survival of HCC patients. In addition, histological differentiation, vascular invasion and TNM stage were also shown to be independent prognostic factors after controlling for all other clinicopathological factors (**Table 2**).

### NDRG2 inhibits LDHA expression, the Warburg effect, and the growth of HCC cells

**Figure 3:**
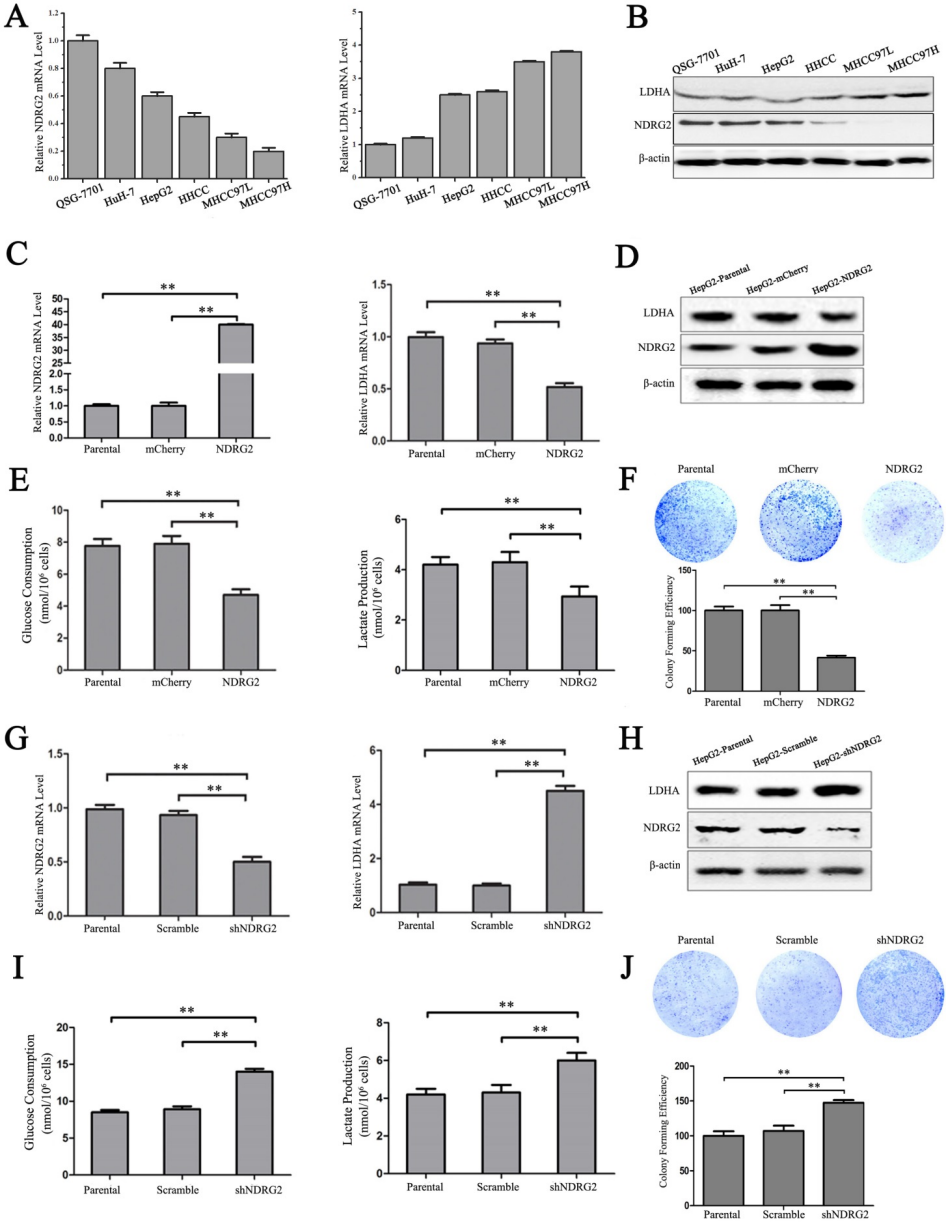
** NDRG2 inhibits LDHA expression and the Warburg effect in HCC cells. (A-B)** NDRG2 and LDHA mRNA and protein levels in HuH-7, HepG2, HHCC, MHCC97L, and MHCC97H HCC cells and in normal QSG-7701 hepatic cells. **(C-E)** NDRG2 and LDHA mRNA levels and protein levels, glucose consumption and lactate production in HepG2 cells that were infected with lentivirus containing NDRG2 or mCherry, and β-actin served as an internal control to ensure equal loading.** (F)** Equal numbers of NDRG2-overexpressing HepG2 cells and control cells were seeded onto a 60-mm dish. After 14 days, the cells were fixed and stained with crystal violet. **(G-I)** NDRG2 and LDHA mRNA levels and protein levels, glucose consumption and lactate production in HepG2 cells that were infected with lentivirus containing NDRG2 shRNA or Scramble, and β-actin served as an internal control to ensure equal loading. **(J)** Equal numbers of NDRG2-knockdown HepG2 cells and control cells were seeded onto a 60-mm dish. After 14 days, the cells were fixed and stained with crystal violet. **p<0.01.

**Figure 4:**
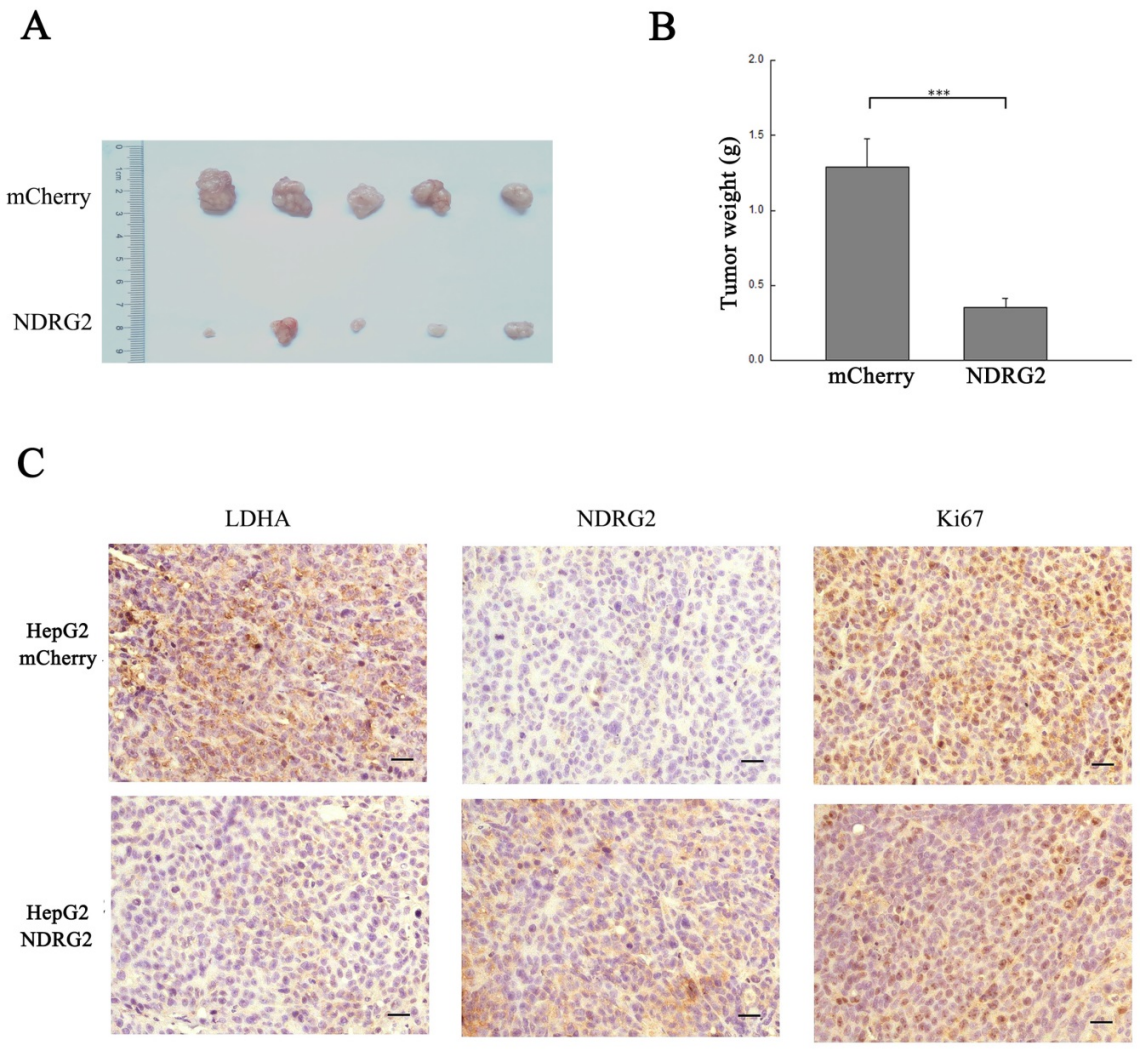
** NDRG2 inhibits the malignant growth and proliferation of HCC cells. (A)** Nude mice were injected subcutaneously in the right limb with 1×10^7^ HepG2 cells or mCherry-overexpressing or NDRG2-overexpressing HepG2 cells respectively. Representative tumor formation was photographed 28 days after injection. **(B)** Tumor weight was calculated at the end of the experiment. **(C)** Immunohistochemical analysis of NDRG2, LDHA and Ki67 antigen expression in tumors of nude mice. The number of Ki67-positive cells was evaluated and compared among the different groups: HepG2, HepG2-mCherry, and HepG2-NDRG2. Scale bars, 30 μm. ***p<0.001.

In addition to the results in the clinical HCC tissues, the expression pattern of NDRG2 had an inverse association with LDHA expression in HCC cell lines. With the decreased differentiation degree and increased metastatic capacity of HCC cancer cell lines, NDRG2 expression decreased while LDHA expression increased (**Figure 3A and 3B**). To identify the underlying molecular mechanism involved in the negative correlation between NDRG2 and LDHA, we analyzed the expression of LDHA in NDRG2-overexpressing and NDRG2-knockdown HepG2 and HuH-7 cells (**Figure S1**). Interestingly, the expression of LDHA decreased in NDRG2-overexpressing HepG2 (**Figure 3C and 3D**) and HuH-7 cells (**Figure S2A and S2C**). Meanwhile, glucose consumption, lactate production and glucose uptake decreased significantly in NDRG2-overexpressing HepG2 cells (**Figure 3E and S3**). The growth and proliferation of cancer cells relies on glycolysis which contributes to ATP production and macromolecule biosynthesis 27. To determine whether NDRG2 regulates the growth and proliferation of HCC cells, plate colony formation assays and *in vivo* tumorigenicity assays were performed. The results of the colony formation assays showed that the growth and proliferation ability also decreased in NDRG2-overexpressing HepG2 cells (**Figure 3F**). Moreover, NDRG2 overexpression inhibited tumor growth *in vivo*. The mice injected with NDRG2-overexpressing HepG2 cells developed tumor slowly than the control groups (**Figure 4A and S4**). By 4^th^ week, mice injected with NDRG2-overexpressing HepG2 cells showed a statistically significant decrease in average tumor weight compared with the control groups (**Figure 4B**). The results of immunohistochemistry staining showed that the number of LDHA and Ki67 positive cells decreased significantly in tumor from nude mice injected with NDRG2-overexpressing HepG2 cells compared with the control groups (**Figure 4C**). Consistent with the changes in NDRG2-overexpressing HepG2 and HuH-7 cells, the expression of LDHA increased significantly in NDRG2-knockdown HepG2 (**Figure 3G and 3H**), NDRG2-knockdown HuH-7 cells **Figure S2B and S2D**). Meanwhile glucose consumption, lactate production, and the growth and proliferation ability increased significantly in NDRG2-knockdown HepG2 cells (**Figure 3I and 3J**). Glucose uptake also increased significantly in NDRG2-knockdown HepG2 cells and NDRG2 knockout MEF cells (**Figure S3**). These results indicated that NDRG2 inhibited LDHA expression and the Warburg effect, and thereby inhibited the malignant growth and proliferation of HCC cells.

### NDRG2 mediates the inhibitory effect of gemcitabine on LDHA expression and Warburg effect in HCC cells

**Figure 5:**
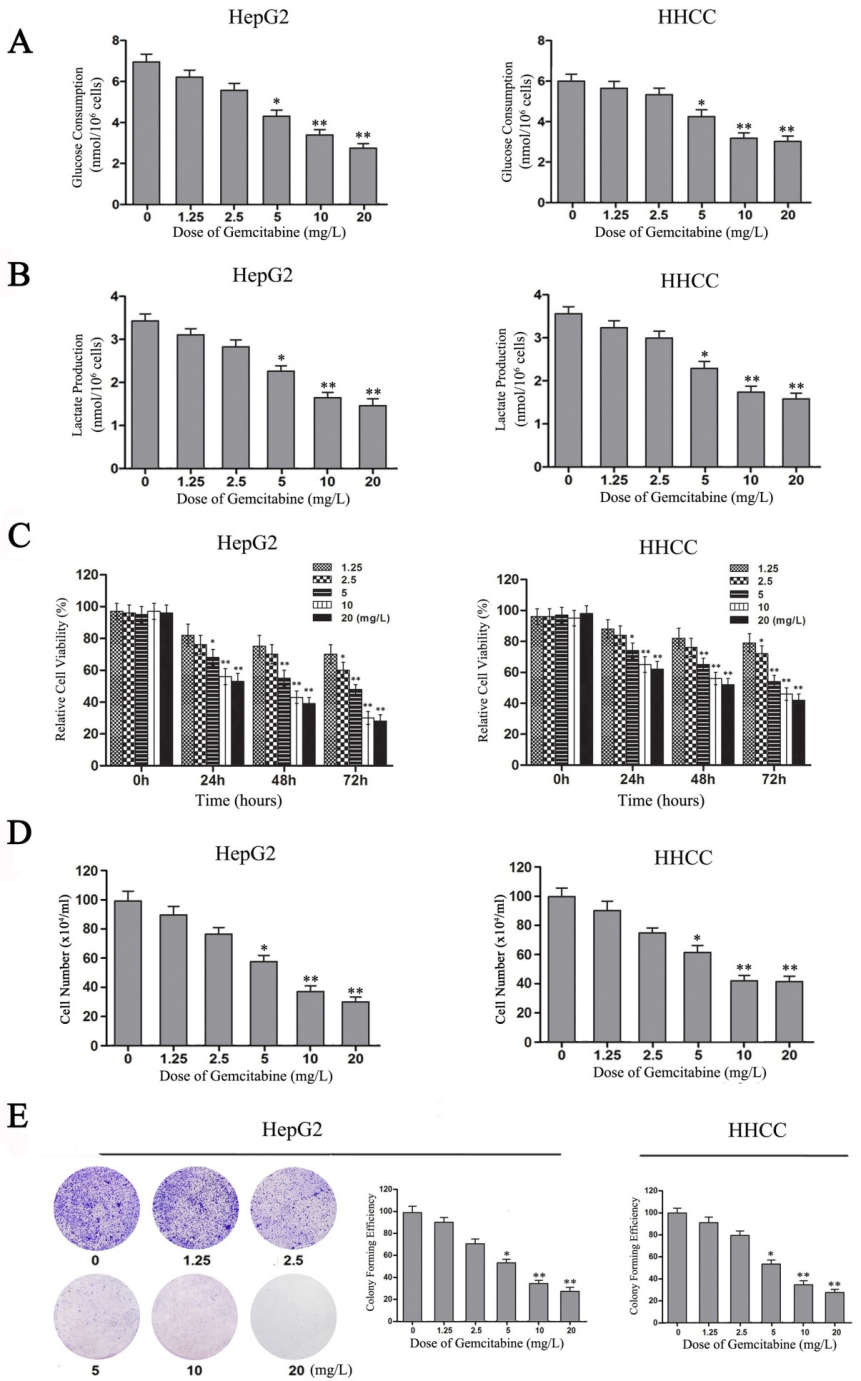
** Gemcitabine inhibits the Warburg effect and the growth of HCC cells. (A-B)** Glucose consumption and lactate production in HepG2 and HHCC cells that were treated with gemcitabine at the indicated concentrations for 24 h. **(C)** HepG2 and HHCC cells were treated with gemcitabine at the indicated concentrations and times, and cell viability was measured with MTT assays. **(D)** HepG2 and HHCC cells were treated with gemcitabine at the indicated concentrations for 48 h, and a cell count analysis was performed. **(E)** Equal numbers of HepG2 cells were seeded onto a 60-mm dish and treated with the indicated concentrations for 14 days, and then, the cells were fixed and stained with crystal violet. All data shown are the mean ± SD of three independent experiments. *p<0.05; **p<0.01.

Gemcitabine is widely applied in combination therapy for advanced HCC 20, 28. Although gemcitabine is considered to be a nucleotide analog that blocks DNA synthesis in tumor cells, our experimental results also showed that gemcitabine can inhibit the Warburg effect in HCC cells. With the increase of gemcitabine concentration, glucose consumption and lactate production decreased in a dose-dependent manner in HepG2 and HHCC cells (**Figure 5A and 5B**). Therefore, gemcitabine can also inhibit aerobic glycolysis in HepG2 and HHCC cells.

To confirm the role of gemcitabine in cell viability, growth and proliferation of HCC cells, MTT and plate colony formation assays were performed to evaluate the viability and growth ability of gemcitabine-treated HCC cells. With the increase of gemcitabine concentration and treatment time, cell viability decreased in a dose-dependent manner compared with the control, especially in the two groups of HCC cells treated with gemcitabine at 10 mg/L and 20 mg/L for 48 h (**Figure 5C**,* P*<0.01). Meanwhile, the results of the living cell count revealed that the number of surviving cells in the gemcitabine-treated HepG2 and HHCC cell populations decreased in a dose-dependent manner with the increase of gemcitabine concentration (**Figure 5D**). Moreover, the colony-forming ability of gemcitabine-treated HepG2 and HHCC cells decreased in a dose-dependent manner with the increase of gemcitabine concentration (**Figure 5E**). Taken together, these results indicated that gemcitabine can suppress the Warburg effect and thereby suppress the cell viability, growth and proliferation ability of HCC cells.

**Figure 6:**
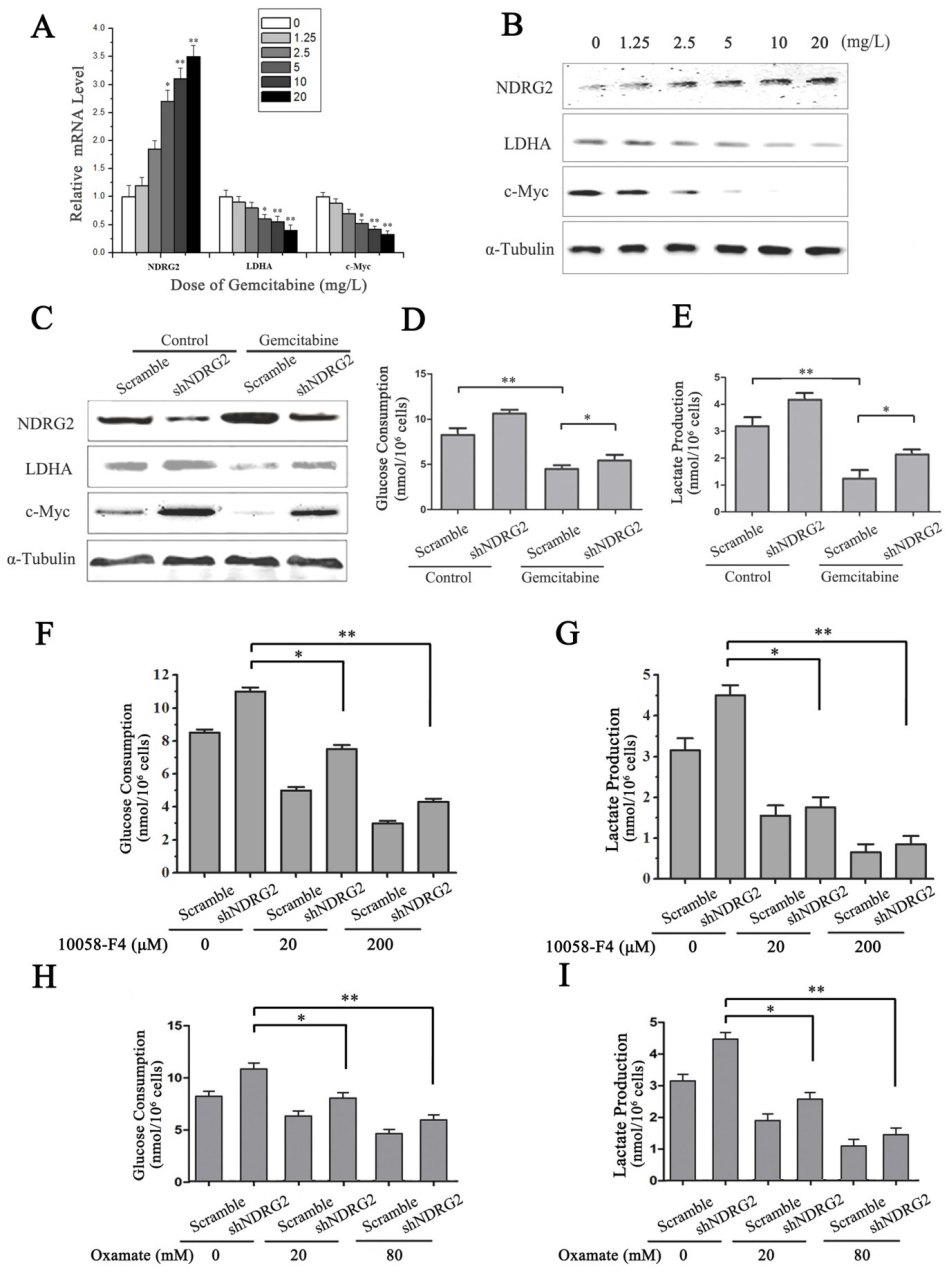
** NDRG2 mediates the inhibitory effect of gemcitabine on Warburg effect in HCC cells. (A-B)** NDRG2, LDHA and C-MYC mRNA and protein levels in HepG2 cells that were treated with gemcitabine at the indicated concentrations for 48 h, and α-tubulin served as an internal control to ensure equal loading. **(C)** LDHA, NDRG2 and c-myc protein levels in HepG2 cells that were infected with lentivirus containing NDRG2 shRNA or Scramble and treated with gemcitabine at 10 mg/L for 48 h, α-tubulin served as an internal control to ensure equal loading. **(D-E)** Glucose consumption and lactate production in HepG2 cells that were infected with lentivirus containing NDRG2 shRNA or Scramble, and treated with gemcitabine at 10 mg/L for 48 h.** (F-G)** Glucose consumption and lactate production in HepG2 cells that were infected with lentivirus containing NDRG2 shRNA or Scramble, and treated with the indicated concentration of c-Myc inhibitor 10058-F4 for 48 h.** (H-I)** Glucose consumption and lactate production in HepG2 cells that were infected with lentivirus containing NDRG2 shRNA or Scramble, and treated with the indicated concentration of oxamate for 48 h. *p<0.05; **p<0.01.

LDHA is an essential enzyme in aerobic glycolysis of cancer cells. LDHA is often overexpressed in cancers and linked to poor prognosis in many cancer lineages 19. In addition, LDHA was significantly suppressed by NDRG2 in colorectal cancer cells and hepatocellular carcinoma cells 13. To identify the underlying target molecules regulated by gemcitabine in HCC aerobic glycolysis, we analyzed LDHA, NDRG2 and c-Myc in HepG2 cells that were treated with gemcitabine at different concentrations. Interestingly, real-time PCR and western blotting analysis showed that NDRG2 expression increased while LDHA and c-Myc expression decreased in a dose-dependent manner with the increase of gemcitabine concentration (**Figure 6A and 6B**). Furthermore, NDRG2 knockdown by shRNA recovered LDHA expression that was suppressed by gemcitabine (**Figure 6C**). Consistent with the changes of LDHA expression in NDRG2-knockdown and gemcitabine-treated HepG2 cells, glucose consumption and lactate production were also recovered in the NDRG2-knockdown HepG2 cells that were treated with gemcitabine (**Figure 6D and 6E**). Interestingly, c-Myc inhibitor 10058-F4 inhibited the enhancement of aerobic glycolysis induced by NDRG2 knockdown in HepG2 cells (**Figure 6F and 6G**). LDHA inhibitor oxamate further inhibited the enhancement of aerobic glycolysis induced by NDRG2 knockdown in HepG2 cells (**Figure 6H and 6I**).

### NDRG2 mediates the inhibitory effect of gemcitabine on malignant growth and tumorigenesis in HCC cells

**Figure 7:**
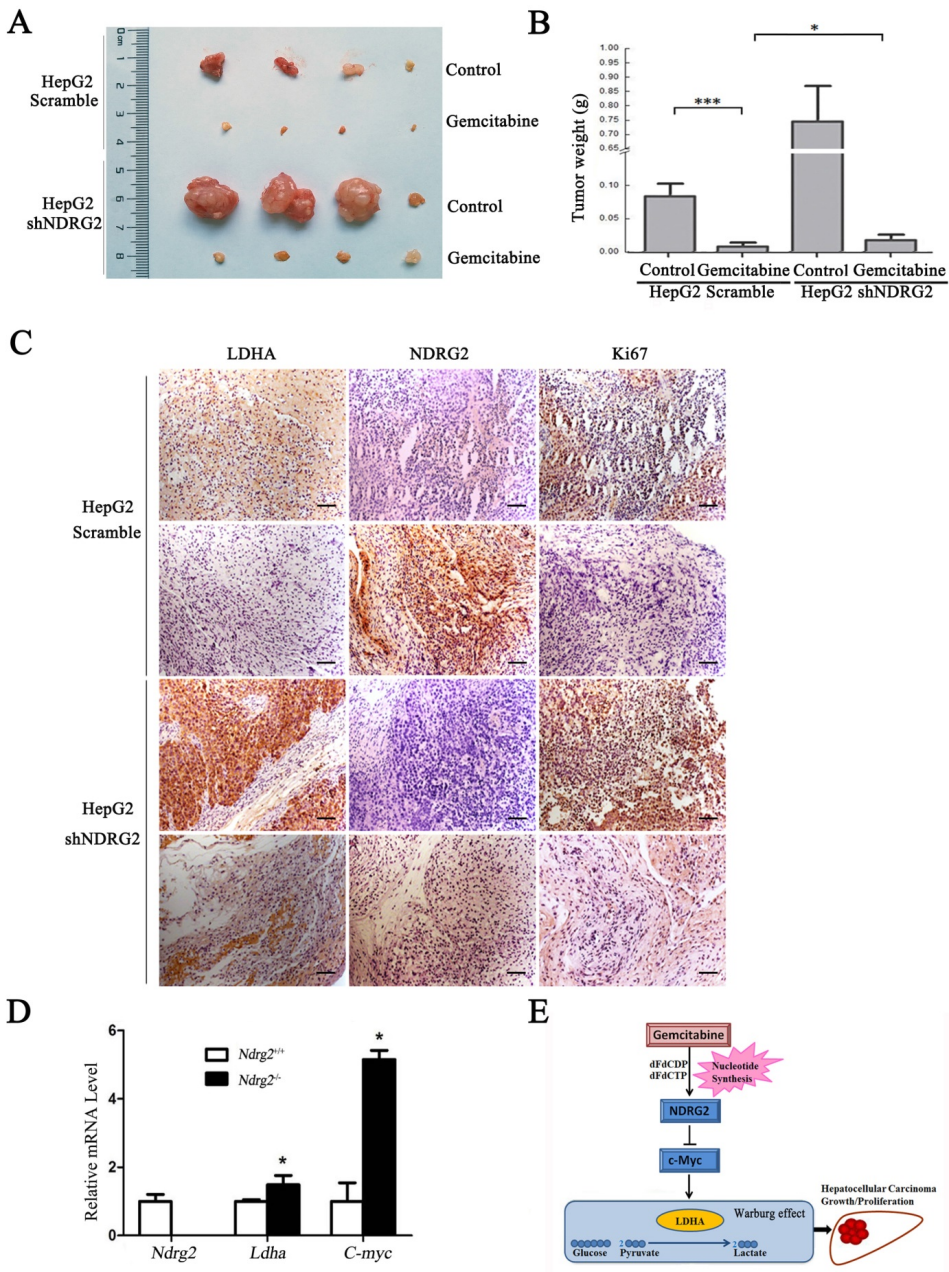
** NDRG2 mediates the inhibitory effect of gemcitabine on malignant growth and tumorigenesis in HCC cells. (A)** Nude mice were injected subcutaneously in the right limb with 1×10^7^ HepG2 that were infected with lentivirus containing NDRG2 shRNA or Scramble. Once the tumor size in nude mice of any group reached an average of 700 mm^3^, the whole mice were treated with 80 mg/kg of gemcitabine diluted in saline on 8, 12 and 16 days post tumor inoculation (d.p.i.). Control mice received saline alone. Representative tumor formation was photographed after the mice were sacrificed.** (B)** Tumor weight was calculated at the end of the experiment.** (C)** Immunohistochemical analysis of NDRG2, LDHA and Ki67 antigen expression in tumors of nude mice. The number of Ki67-positive cells was evaluated and compared among the different groups: HepG2-Scramble+saline, HepG2 Scramble+gemcitabine, HepG2-shNDRG2+saline, and HepG2-shNDRG2+gemcitabine. Scale bars, 30 μm. **(D)** *Ndrg2*, *Ldha* and *C-myc* mRNA levels in *Ndrg2* knockout Murine Embryonic Fibroblasts (MEF) cells were analyzed by quantitative real-time PCR. **(E)** A suggested model for the regulation of metabolic pathways in hepatocellular carcinoma cells by the tumor suppressor NDRG2, which mediates the effect of the chemotherapeutic drug gemcitabine. Scale bars, 30 μm. *p<0.05; ***p<0.001.

To determine whether NDRG2 mediate the inhibitory effect of gemcitabine on the growth and proliferation of HCC cells, *in vivo* tumorigenicity assay was performed. We established xenograft tumors using HepG2 cells expressing either NDRG2 shRNA or scramble shRNA. Once tumor was eatablished, mice were treated with gemcitabine, and tumor weight was measured. As expected, gemcitabine inhibited tumor growth *in vivo*, and NDRG2 knockdown partly recovered the malignant tumor growth and proliferation in nude mice that was suppressed by gemcitabine (**Figure 7A, 7B and S5**). Immunohistochemistry staining also conformed Ki67 and LDHA expression recovered in mice harboring tumors with NDRG2-knockdown HepG2 cells treated with gemcitabine (**Figure 7C**). In addition, the mRNA levels of *Ldha* and *C-myc* were upregulated in *Ndrg2* knockout MEF cells. (**Figure 7D**). Therefore, LDHA is a key mediator of Warburg effect in NDRG2-loss HCC cells. These data demonstrate that gemcitabine inhibited LDHA expression and the Warburg effect, and these effects were mediated by NDRG2 in HCC cells (**Figure 7E**). Thus, our study suggested that NDRG2 could be a potential novel prognostic marker and a new therapeutic target to improve the treatment and survival of HCC patients.

## Discussion

Hepatocellular carcinoma (HCC) is one of the most common malignant tumors, and it ranks as the third most common cause of cancer-related death worldwide 29. In the last decades, the incidence of HCC has been increasing significantly 30. Due to late diagnosis and advanced underlying cirrhosis, only limited treatments to delay and block HCC progression are available. Therefore, patients with advanced HCC have a very low level of survival 31. Research on molecular alterations and clinical outcome is vital in HCC research. One of the greatest challenges in HCC treatment is to accurately predict relapse and outcome for each patient to determine who will receive appropriate treatment strategies. Currently, clinical doctors rely heavily on traditional pathologic variables, and the TNM staging system of HCC is an important standard for determining prognosis in patients with HCC. However, even patients with the same TNM stage of HCC show differences in survival. Therefore, identification of more molecules that are involved in HCC relapse and prognosis might be of great significance to discover new biomarkers for early detection and therapeutic targets.

N-Myc downstream-regulated gene 2 (NDRG2) belongs to the NDRG family and acts as a tumor suppressor gene. We previously reported that NDRG2 is widely expressed in many normal tissues and is decreased in many types of tumor tissues 6, 26, 32-35. Moreover, NDRG2 also inhibited the growth and proliferation ability of tumor cells as well as induced apoptosis and differentiation of HCC cells and other types of tumor cells 6, 7, 36, 37. In addition to malignant growth and invasion, metabolic abnormality is considered as the new phenotype of cancer cells 38. The tumor suppressor NDRG2 also plays an important role in tumor metabolic reprogramming. In colorectal cancer cells, we discovered that NDRG2 inhibits glycolysis and glutaminolysis 13. In hepatocellular carcinoma cells with active metabolism, NDRG2 also inhibits aerobic glycolysis, cell growth, cell proliferation and mediates the inhibitory effect of gemcitabine on aerobic glycolysis.

The enzyme LDH catalyzes the conversion of pyruvate to lactate under aerobic conditions and mediates aerobic glycolysis. Persistent aerobic glycolysis is a key metabolic dependency in tumorigenesis. Unlike other subunits, LDHA is primarily expressed in cancer cells and contributes to tumorigenesis 16. LDHA expression is closely related with the survival outcomes of patients 39. Furthermore, attenuation of LDHA expression resulted in the decrease of aerobic glycolysis and stimulation of mitochondrial respiration in tumor cells, and also compromised the ability of these tumor cells to proliferate under hypoxia 40. Therefore, LDHA is a key mediator of Warburg effect of tumor cells. The expression of LDHA can be regulated by many factors. The transcriptional factors c-Myc and HIF-1 can transcriptionally regulate LDHA alone or in collaboration with each other 41-43. The PI3K/Akt/mTOR pathway modulates HIF-1α protein synthesis, and thereby activates LDHA expression 44. Furthermore, the pyruvate kinase isozyme M2 (PKM2) acts as a transcriptional coactivator that interacts with HIF-1α and promotes transactivation of HIF-1 target gene LDHA in cancer cells 45, 46. In addition, ErbB2 can upregulate LDHA expression through heat shock factor 1 47. Our preliminary results showed that c-Myc may be involved in the regulation of LDHA expression induced by NDRG2.

**Figure 8:**
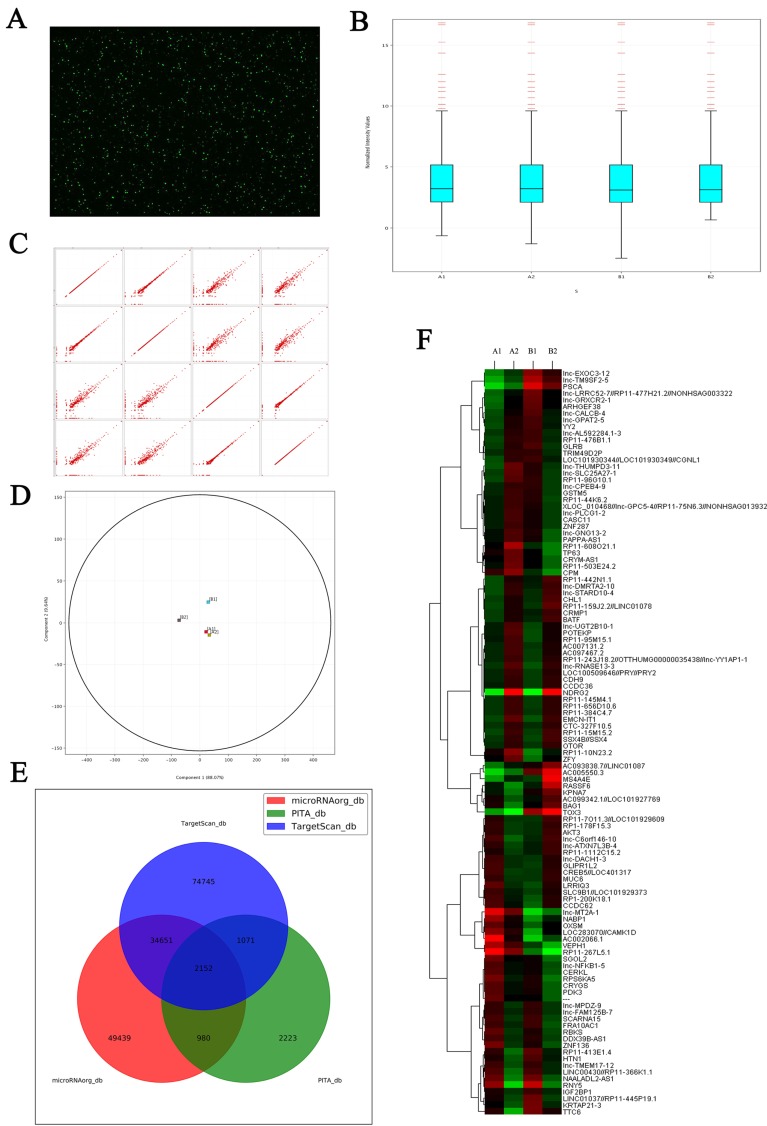
** Microarray shows the changes of LncRNAs expression induced by NDRG2 overexpression in colorectal carcinoma cell. (A)** Total RNAs extracted from treated cells were transcribed into double strand cDNAs and then synthesized cRNAs. Next, 2^nd^ cycle cDNAs were synthesized from cRNAs. Followed fragmentation and biotin labeling, the 2^nd^ cycle cDNAs were hybridized onto the microarray. After washing and staining, the arrays were scanned by the Affymetrix Scanner 3000. **(B)** Box-whisker plot presents the normalized microarray expression data.** (C)** Scatter plot shows the log expression ratios of microarray expression data. **(D)** Principal component analysis shows the distribution of microarray expression data.** (E)** Prediction for differential expression of miRNAs between NDRG2 overexpression and control groups using TargetScan, PITA, microRNAorg database. **(F)** Heat map shows miRNAs that expresses difference between NDRG2 overexpression and control groups. The relative fold change for each miRNA in each sample is represented as a relative mean value increase (red) or decrease (green). A1: mCherry-overexpressing HCT116 cells, A2: NDRG2-overexpressing HCT116 cells, B1: mCherry-overexpressing HT29 cells, B2: NDRG2-overexpressing HT29 cells.

In this current study, we first observed the correlation between NDRG2 and LDHA expression in HCC patients and the prediction of HCC prognosis based on NDRG2 and LDHA expression. Our results showed that NDRG2 and LDHA expression correlated with histological differentiation, vascular invasion and TNM stage of HCC patients, but not correlated with tumor size of HCC patients. This is possibly attributed to the source of HCC tissues from the patients undergoing hepatic resection. The patients with solitary small HCC were mainly treated with radiofrequency ablation (RFA) and transarterial chemoembolization (TACE). Meanwhile, the patients with huge HCC and extrahepatic metastasis have to give up hepatic resection. The tumor size of HCC tissues from the patients undergoing hepatic resection is relatively moderate, and therefore not correlated closely with NDRG2 and LDHA expression. In addition, NDRG2 expression had an inverse association with LDHA expression and NDRG2 inhibited LDHA expression in hepatocellular carcinoma. There results were consistent with our previous study in colorectal carcinoma, in which we found that NDRG2 inhibited LDHA expression and aerobic glycolysis in colorectal carcinoma by repressing c-Myc expression. Since NDRG2 was mainly located in cell cytoplasm and regulated LDHA expression indirectly, c-Myc knockdown attenuated the inhibitory effect of NDRG2 on LDHA expression 13. Since c-Myc mediated the inhibitory effect of NDRG2 on LDHA expression, our research group further discovered that NDRG2 repressed the expression of c-Myc at the transcriptional level by inhibiting the expression of β-catenin 13. It is well documented that nuclear β-catenin binds to T-cell factor/lymphoid enhancer factor (TCF/LEF) and thereby activates transcription of its target genes such as c-Myc 48. Therefore, NDRG2 suppressed c-Myc expression by inhibiting the expression of β-catenin. As NDRG2 is mainly located in cell cytoplasm, the related regulation mechanism by which NDRG2 effects β-catenin depends on molecules regulated by NDRG2. LncRNAs regulated by NDRG2 have been screened by microarrays and LncRNAs RP11 are regulated by NDRG2. For example, RP11-145M4.1, RP11-656D10.6, RP11-384C4.7, RP11-10N23.2 is upregulated by NDRG2 and RP11-366K1.1, RP11-455P19.1 is downregulated by NDRG2 (**Figure 8**). Whether these LncRNAs are related to the regulation of LDHA by NDRG2 is being investigated.

Gemcitabine is an effective anticancer chemotherapeutic drug that is widely used against solid tumors 49. Though the pharmacological mechanism of gemcitabine is attributed to the fact that it is an analogue of natural nucleotides and thereby disrupts nucleic acid synthesis, our research results showed that gemcitabine inhibited LDHA expression and aerobic glycolysis of HCC cells. Interestingly, NDRG2 is upregulated in gemcitabine-treated HCC cells, and it mediated the inhibitory effect on LDHA expression and aerobic glycolysis induced by gemcitabine. Gemcitabine is an analog of cytosine arabinoside which has produced remarkable radiosensitization in a variety of solid tumors and tumor cell lines. It is reported that gemcitabine induced the upregulation of p53 in normal p53 function cancer cells 50. We also observed the mRNA level of p53 increased in gemcitabine treated HepG2 cells by real-time PCR. More importantly, p53 can transcriptionally activate NDRG2 gene which is involved in the p53-mediated apoptosis pathway 7. Maybe the upregulation of NDRG2 induced by gemcitabine attributes to the upregulation of p53 and inhibition of nucleic acid synthesis, but the molecular mechanism still needs to be further investigated.

In summary, this study analyzed and provided the first evidence that the expression level of NDRG2 and LDHA in HCC might be powerful predictors of disease relapse and prognosis. NDRG2 inhibited LDHA expression and thereby inhibited aerobic glycolysis, growth and proliferation of HCC cells. Furthermore, the chemotherapeutic drug gemcitabine inhibited aerobic glycolysis, growth and proliferation of HCC cells. NDRG2 mediated this inhibitory effect of gemcitabine on LDHA expression and aerobic glycolysis in HCC cells. Although further study will be needed to explore the regulatory role and molecular mechanism of NDRG2 in modulation of LDHA expression and aerobic glycolysis of HCC cells, our present findings support the notion that NDRG2, combined with LDHA, might be valuable markers to evaluate the prognosis of HCC patients. Meanwhile, NDRG2 might also be a potential therapeutic target for molecular targeted therapy of HCC.

## Supplementary Material

Supplementary figures and tables.

## Figures and Tables

**Table 1:** Associations of NDRG2 and LDHA expression with clinical factors in patients with HCC

Variables	NDRG2			LDHA		
	Low (n=87)	High (n= 53)	*P* value	Low (n=48)	High (n=92)	*P* value
Age(y)			0.220			0.428
≤60	59	40		33	66	
>60	28	13		15	26	
Gender			0.379			0.216
Male	69	44		41	72	
Female	18	9		7	20	
AFP(ng/ml)			0.113			0.327
≤400	32	28		23	39	
>400	53	27		25	53	
Tumor size(cm)			0.231			0.135
Small(≤5)	49	34		32	51	
Large(>5)	38	19		16	41	
Histological differentiation			< 0.001			< 0.001
Well	9	18		20	7	
Moderate	54	20		20	54	
Poor	24	15		8	31	
Vascular invasion			< 0.001			0.024
Absent	45	43		36	52	
Present	42	10		12	40	
TNM stage			< 0.001			0.027
Ⅰ	25	31		25	31	
Ⅱ-Ⅲ	62	22		23	61	
HBV			0.534			0.433
Absent	14	9		7	16	
Present	73	44		41	76	

TNM stage for HCC was based on The American Joint Committee on Cancer/International Union Against Cancer staging system.

**Table 2:** Univariate and Multivariate Cox regression analysis of prognostic factors for overall survival in 140 patients with HCC

Clinical variables	Univariate analysis			Multivariate analysis		
	HR	95% CI	*P* value	HR	95% CI	*P* value
Age (≥60 vs. <60)	1.184	0.957-2.617	0.379			
Gender (Male vs. Female)	0.767	0.554-1.234	0.407			
AFP (≥400 ng/ml vs. <400 ng/ml)	1.153	0.969-1.723	0.493			
Tumor size (≥5 cm vs. <5cm)	2.001	0.861-4.649	0.107			
Histological differentiation (Poor vs. Well)	1.554	1.020-2.370	0.040	1.842	1.104-3.072	0.019
Vascular invasion (Present vs. Absent)	2.464	1.395-3.650	0.001	2.698	1.587-3.648	0.003
TNM stage (II-III vs. I)	2.674	1.528-4.680	0.001	2.514	1.391-4.544	0.002
HBsAg (Positive vs. Negative)	0.663	0.356-1.236	0.196			
NDRG2 (Low vs. High)	2.934	1.532-5.433	0.001	2.596	1.332-5.062	0.006
LDHA (High vs. Low)	2.452	1.393-4.575	0.005	2.145	1.134-4.186	0.029

**Abbreviations:** HR, hazard ratio; CI, confidence interval

## Abbreviations

NDRG2: N-myc downstream regulated gene 2
LDHA: lactate dehydrogenase A
HCC: hepatocellular carcinoma
TMA: tissue microarray
PKM2: pyruvate kinase M2
TCF/LEF: T-cell factor/lymphoid enhancer factor
MEF: murine embryonic fibroblasts
shRNA: small hairpin RNA
