# Supplementary material for: Combined Aberrant Expression of NDRG2 and LDHA Predicts Hepatocellular Carcinoma Prognosis and Mediates the Anti-tumor Effect of Gemcitabine
Source: Int J Biol Sci. 2019 Jul 3;15(9):1771–86. doi: 10.7150/ijbs.35094 (PMC6743297; doi:10.7150/ijbs.35094)
Supplement: Supplementary file 1 — Supplementary figures and tables. [file ijbsv15p1771s1.pdf]

Supplementary Information

Combined Aberrant Expression of NDRG2 and LDHA Predicts Hepatocellular Carcinoma Prognosis and Mediates the Anti-tumor Effect of Gemcitabine

Yan Guo[1*], Xi'an Li[1*], Xiang Sun[2*], Jiancai Wang[1], Xu Yang[1], Xin Zhou[1], Xinping Liu[1], Wenchao Liu[3], Jianlin Yuan[4], Libo Yao[1], Xia Li[1], Lan Shen[1✉]

1. The State Key Laboratory of Cancer Biology, Department of Biochemistry and Molecular Biology, The Fourth Military Medical University, Xi'an, Shaanxi, 710032, China;
2. Department of Prosthodontics, School of Stomatology, The Fourth Military Medical University, Xi'an, Shaanxi, 710032, China
3. Department of Oncology, State Key Discipline of Cell Biology, Xijing Hospital, The Fourth Military Medical University, Xi'an, Shaanxi, 710032, China;
4. Department of Urology, Xijing Hospital, The Fourth Military Medical University, Xi'an, Shaanxi, 710032, China;

[*] These authors contributed equally to this work.

✉ Corresponding author: Lan Shen, The State Key Laboratory of Cancer Biology, Department of Biochemistry and Molecular Biology, The Fourth Military Medical University, Xi'an Shaanxi, 710032, China. Tel: +86-029-84774513; E-mail: lanshen@fmmu.edu.cn

Running title: NDRG2 inhibits LDHA expression and mediates the pharmacological effect of gemcitabine

Supplementary Data

Supplementary Figures and Figure legends

Supplementary Figure 1

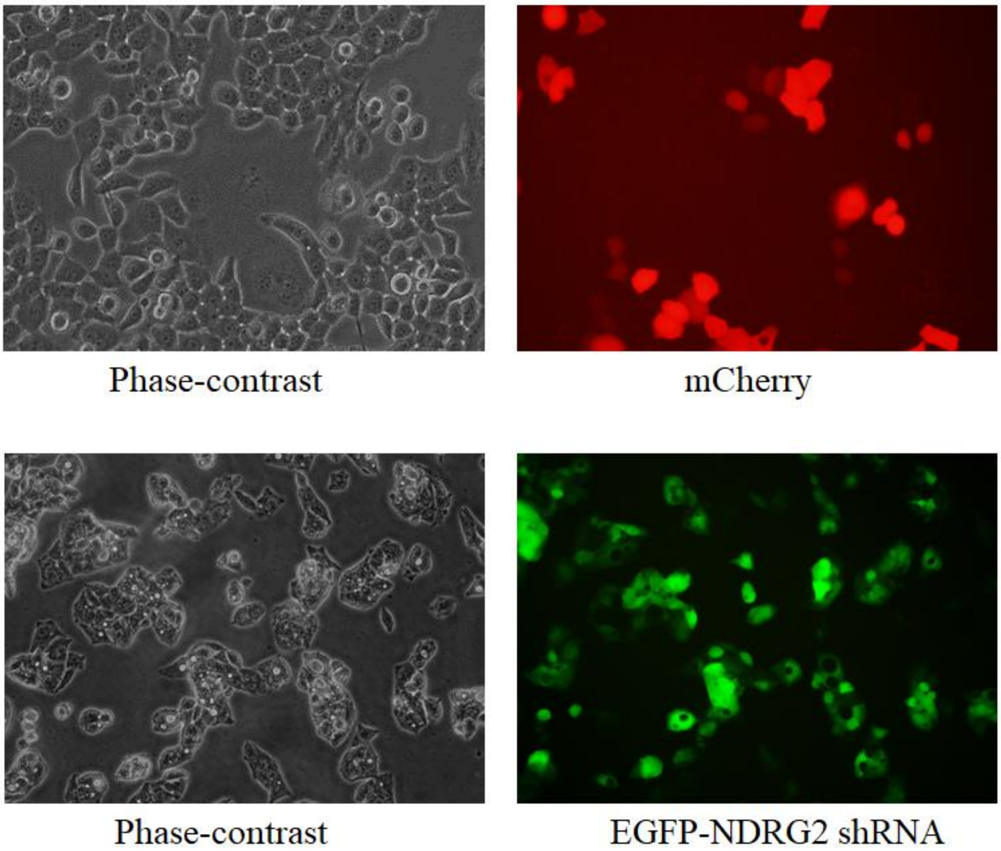

Supplementary Figure 1. Fluorescence microscopy shows the infection efficiency of HepG2 cells

HepG2 cells were infected with Lenti-mCherry or Lenti-EGFP-NDRG2 shRNA for 48 h and then imaged with confocal fluorescence microscopy.

Supplementary Figure 2

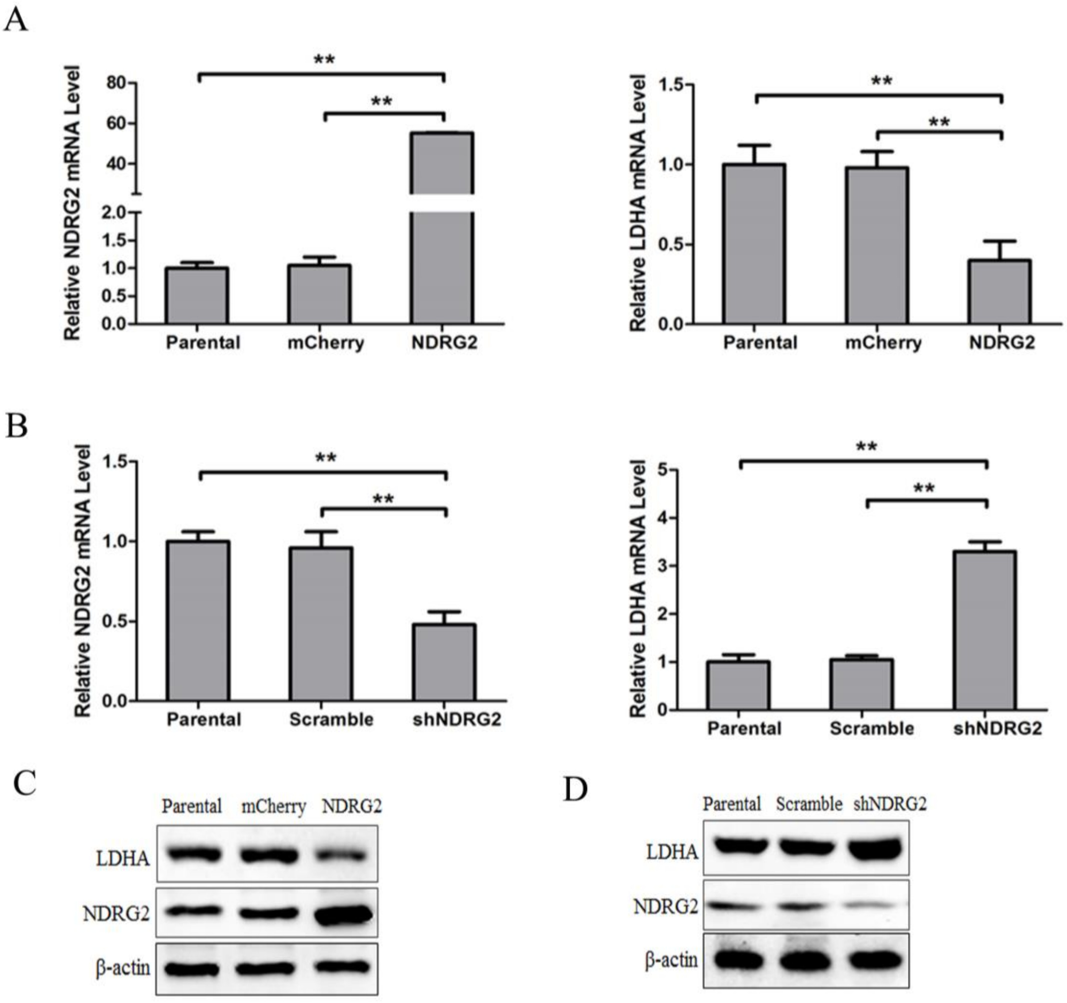

Supplementary Figure 2. NDRG2 inhibits LDHA expression in HuH-7 cells

(A) NDRG2 and LDHA mRNA levels in HuH-7 cells that were infected with lentivirus containing NDRG2 or mCherry, and β-actin served as an internal control to ensure equal loading. **(B)** NDRG2 and LDHA mRNA levels in HuH-7 cells that were infected with lentivirus containing NDRG2 shRNA or Scramble, and β-actin served as an internal control to ensure equal loading. **(C)** NDRG2 and LDHA protein levels in HuH-7 cells that were infected with lentivirus containing NDRG2 or mCherry, and β-actin served as an internal control to ensure equal loading. **(D)** NDRG2 and LDHA protein levels in HuH-7 cells that were infected with lentivirus containing NDRG2 shRNA or Scramble, and β-actin served as an internal control to ensure equal loading. All $**P<0.01$.

Supplementary Figure 3

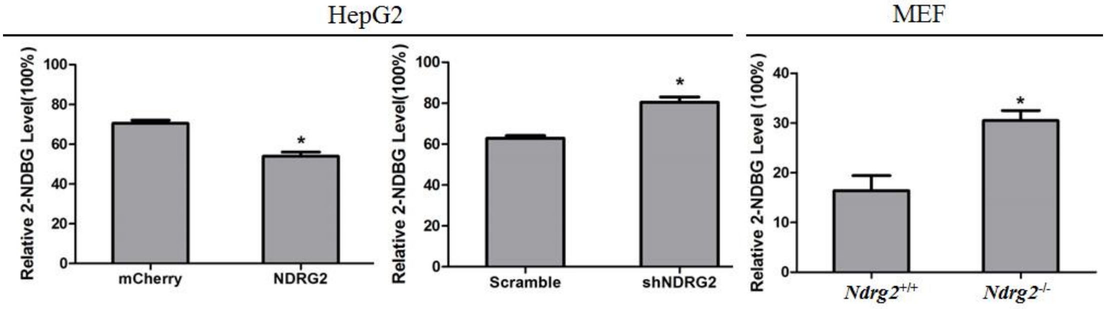

Supplementary Figure 3. NDRG2 inhibits glucose uptake in HepG2 and MEF cells

Glucose uptake in HepG2 cells that were infected with lentivirus containing NDRG2 or mCherry (left), NDRG2 shRNA or Scramble (middle), and *Ndrg2* knockout Murine Embryonic Fibroblasts (MEF) cells (right). All *$P<0.05$.

Supplementary Figure 4

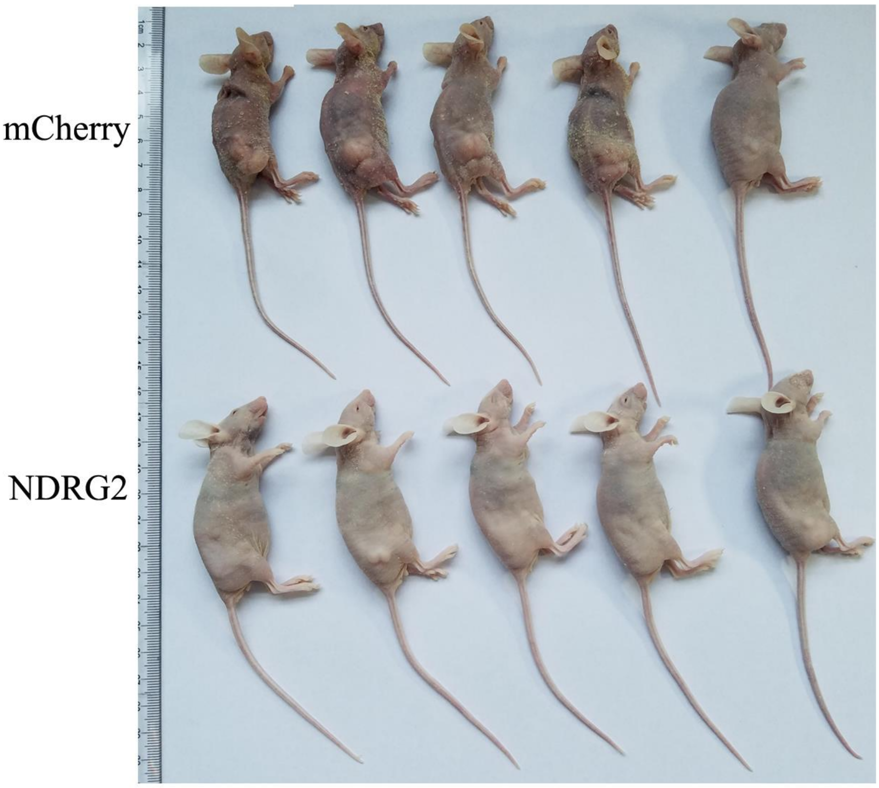

Supplementary Figure 4. NDRG2 inhibits the growth and proliferation of HCC cells

Nude mice were injected subcutaneously in the right limb with 1×10^7 HepG2 cells or mCherry-overexpressing or NDRG2-overexpressing HepG2 cells respectively. Representative tumor formation was photographed 28 days after injection.

Supplementary Figure 5

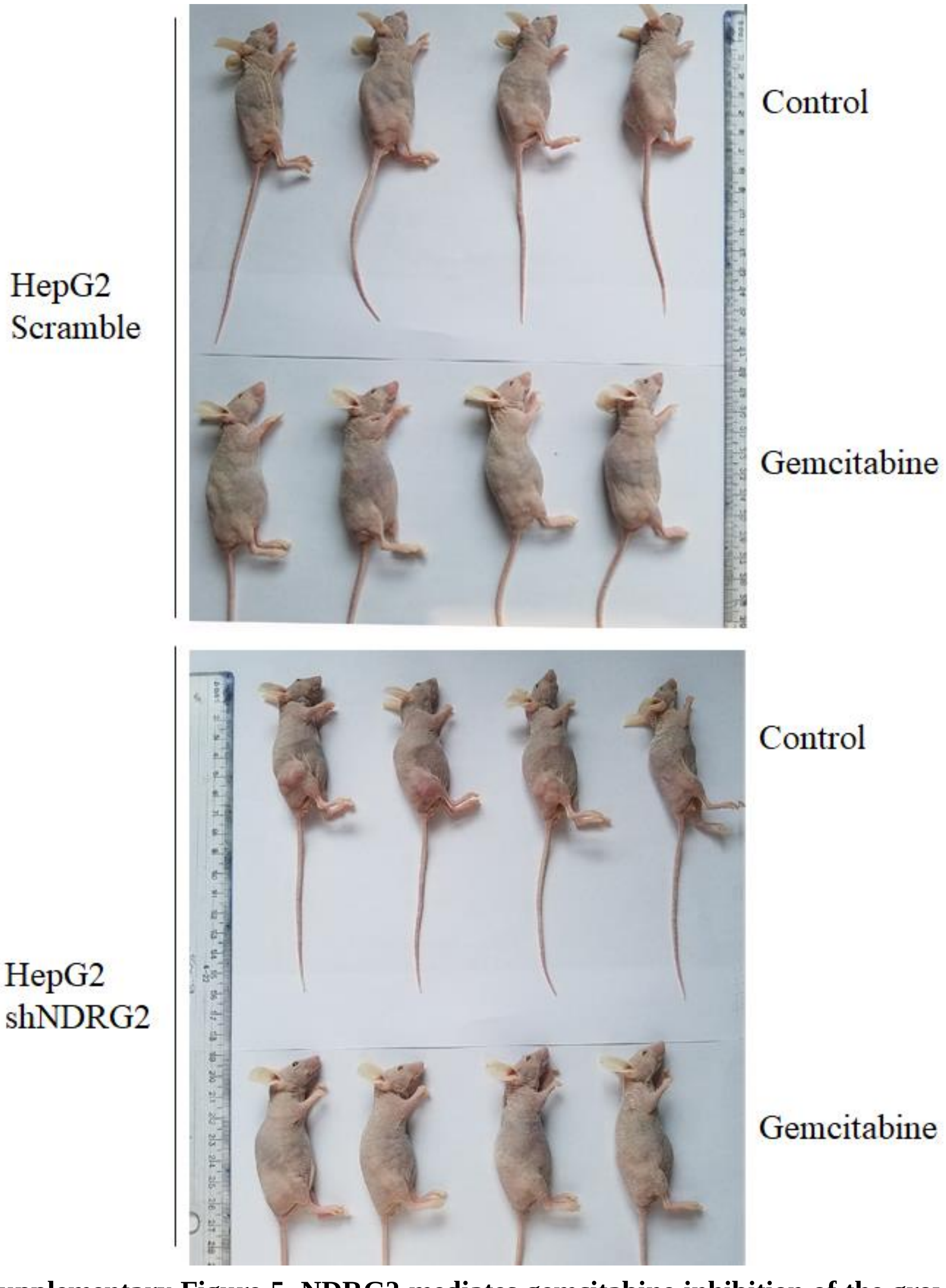

Supplementary Figure 5. NDRG2 mediates gemcitabine inhibition of the growth of HCC cells

Nude mice were injected subcutaneously in the right limb with 1×10^7 HepG2 that were infected with lentivirus containing NDRG2 shRNA or Scramble. Once the tumor size reached an average of 700 mm^3, the mice were treated with 80 mg/kg of gemcitabine diluted in saline on 8, 12 and 16 days post tumor inoculation (d.p.i.).. Control mice received saline alone. Representative tumor formation was photographed after injection.

Supplementary Table

Supplementary Table 1. The primers used for quantitative real-time RT-PCR

Gene	Forward	Reverse
NDRG2	5’-GAGATATGCTCTTAACCACCCG-3’	5’-GCTGCCCAATCCATCCAA-3’
LDHA	5’-CTGGGAGTTCACCCATTAAGCT-3’	5’-CAGGCACACTGGAATCTCCAT-3’
C-MYC	5’-CAGTGGGCTGTGAGGAGGTT-3’	5’-CAGGCTCCTGGCAAAAGGT-3’
β-ACTIN	5’-CGCGAGAAGATGACCCAGAT-3’	5’-GTACGGCCAGAGGCGTACAG-3’
Ndrg2	5’-ACACCTTATGGCTCGGTCAC-3’	5’-TCTCTTGCATATCCCCGAAC-3’
Ldha	5’-CAAAGACTACTGTGTAACTGCGA-3’	5’-TGGACTGTACTTGACAATGTTGG-3’
C-myc	5’-GCTCGCCCAAATCCTGTA-3’	5’-TCCACAGACACCACATCAA-3’
β-Actin	5’-CGTGAAAAGATGACCCAGAT-3’	5’-GTACGACCAGAGGCATACAG-3’
